# Contact forces in roughness discrimination

**DOI:** 10.1038/s41598-020-61943-x

**Published:** 2020-03-20

**Authors:** Roberta D. Roberts, Aldrin R. Loomes, Harriet A. Allen, Massimiliano Di Luca, Alan M. Wing

**Affiliations:** 1 School of Psychology, University of Birmingham, Birmingham, B15 2TT United Kingdom; 2 School of Psychology, University of Nottingham, Nottingham, NG7 2RD United Kingdom

**Keywords:** Sensory processing, Human behaviour

## Abstract

Roughness perception through fingertip contact with a textured surface can involve spatial and temporal cues from skin indentation and vibration respectively. Both types of cue may be affected by contact forces when feeling a surface and we ask whether, on a given trial, discrimination performance relates to contact forces. We examine roughness discrimination performance in a standard psychophysical method (2-interval forced choice, in which the participant identifies which of two spatial textures formed by parallel grooves feels rougher) while continuously measuring the normal and tangential forces applied by the index finger. Fourteen participants discriminated spatial gratings in fine (spatial period of 320–580 micron) and coarse (1520–1920 micron) ranges using static pressing or sliding contact of the index finger. Normal contact force (mean and variability) during pressing or sliding had relatively little impact on accuracy of roughness judgments except when pressing on surfaces in the coarse range. Discrimination was better for sliding than pressing in the fine but not the coarse range. In contrast, tangential force fluctuations during sliding were strongly related to roughness judgment accuracy.

## Introduction

The microgeometry of surfaces, such as the spatial periodicity in the grain of planed wood or the random periodicity of sandpaper, is called texture and is the major determinant of perceived roughness. When judging surface roughness, people typically press the finger pad into the surface or slide it across the surface^1,2^. Pressing indents the skin with the surface topography which excites slowly adapting (SA) tactile mechanoreceptors^3^. Sliding sets up skin vibrations which excite rapidly adapting Pacinian (PC) mechanoreceptors, but may also stimulate spindles of muscles mechanically linked to the finger-tip^4–6^. Together, SA and PC mechanoreceptors provide spatial and vibration cues. The duplex theory of roughness perception^4,7–10^ proposes that fine spatial textures (spatial dimensions up to a few hundred microns) are discriminated in terms of vibration during sliding, whereas coarse textures (spatial dimensions of several hundreds or thousands of microns) are discriminated in terms of spatial pattern during pressing.

Vibration and spatial indentation are both likely to be affected by contact forces during pressing or sliding which might affect roughness perception. Moreover, given differing cues to roughness from pressing or sliding the finger over a surface, it might be expected that contact forces normal and tangential to the surface might have contrasting effects in pressing and sliding on any relation with roughness discrimination accuracy. Previous studies have assessed whether contact force normal to the surface affects roughness magnitude judgments^11–15^ or roughness discrimination^16,17^. The surfaces used in these studies have included spatial gratings defined by grooves and ridges, raised dot or truncated cone arrays and sandpaper samples. The form of touch has been limited to sliding contact between the finger and the stimulus. Using a magnitude estimation task, Lederman and Taylor^13^ showed perceived roughness increases with greater normal force of the finger actively sliding across periodic gratings. Smith *et al*.^14^ failed to replicate this effect of normal force, however, they observed fluctuations in tangential force during active sliding whose power increased with magnitude estimates of the roughness of truncated cones. In a discrimination task, Lamb^18^ found no effect of a near doubling of normal force on the roughness threshold of raised dot arrays passively scanned across the index finger. Libouton *et al*.^16^ found no correlation between roughness thresholds and normal force used in actively scanning the index finger across sandpapers of varying grit size.

The preceding studies of force effects on roughness perception covered a range of tasks and stimulus materials. However, none of these studies determined whether there are systematic changes in contact forces with sliding vs pressing and as a function of fine or coarse levels of roughness nor whether trial to trial differences in contact force parameters are related to the accuracy of roughness discrimination. In this paper we examine the relation between normal force (during fingertip pressing) and normal and tangential force (during fingertip sliding) and roughness discrimination performance in a 2-interval forced choice (2-IFC) paradigm where each trial includes a standard and a comparison stimulus (in random order). To provide stimuli varying in roughness we use pairs of periodic spatial gratings in either fine (320–580 micron) or coarse (1520–1920 micron) ranges. We examine whether, on a trial by trial basis, discrimination performance (correct vs incorrect response) is related to the contact force parameters on that trial. The parameters considered include: mean and within-trial variability of normal and tangential force and differences in normal and tangential force when touching the two stimuli making up a trial. Based on the duplex theory contrast between spatial and temporal cues to roughness, we predict a relation that depends on the coarseness of the texture and type of movement. For coarse textures we expect spatial cues should be more informative. Thus, in pressing against a coarse texture, we predict trials with greater normal force should lead to improved discrimination performance as the spatial topography of the texture on the skin will be better registered. For fine textures, we expect vibration cues should be more informative. Thus, in sliding over fine textures, we predict better discrimination between trials with more difference in tangential force variability (which is an index of vibratory information) between the pair of touched surfaces.

## Results

**Figure 1:**
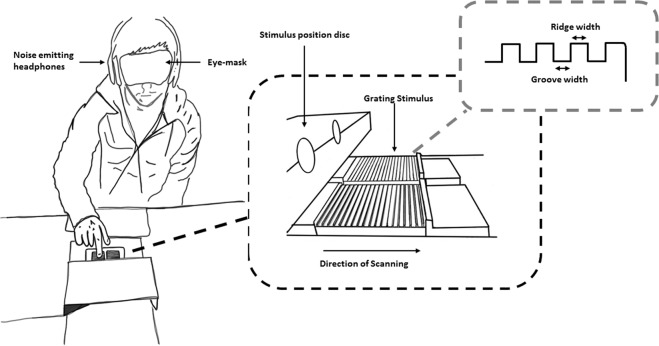
Experimental set up. Stimulus gratings are located in a metal casing mounted on a 6 degree of freedom force-torque sensor. Before stroking each grating (moving the finger in a line towards the body) they lightly touched a small metal locating dome at the end of the grating). Participants wore a blindfold and headphones playing white noise to mask visual and auditory cues to grating roughness.

14 participants took part in the experiment and explored two surfaces in close succession (Fig. 1), a standard (groove widths 220 or 1120 µm) and a comparison chosen at random in the matching range (groove widths 320–480 or 1200–1520 µm) (see Table 1). The order of the standard and comparison was randomised on a trial-by-trial basis. Alpha of 0.05 was set as the criterion for statistical significance.

**Table 1:** The details of surfaces used in the roughness judgment task.

Ridge Width		Groove Width					
		*Standard Stimulus (μm)*	*Comparison Stimulus (μm)*				
			Least Coarse			Most Coarse	
Fine Surfaces	100	220	320	360	400	440	480
Coarse Surfaces	400	1120	1200	1280	1360	1440	1520

Ridge and groove widths of the square wave surfaces are shown in microns for a high spatial frequency group (Fine Surfaces) and a lower frequency group (Coarse Surfaces). Microscopic inspection revealed GW’s to be within 6% of target values.

### Roughness judgments

**Figure 2:**
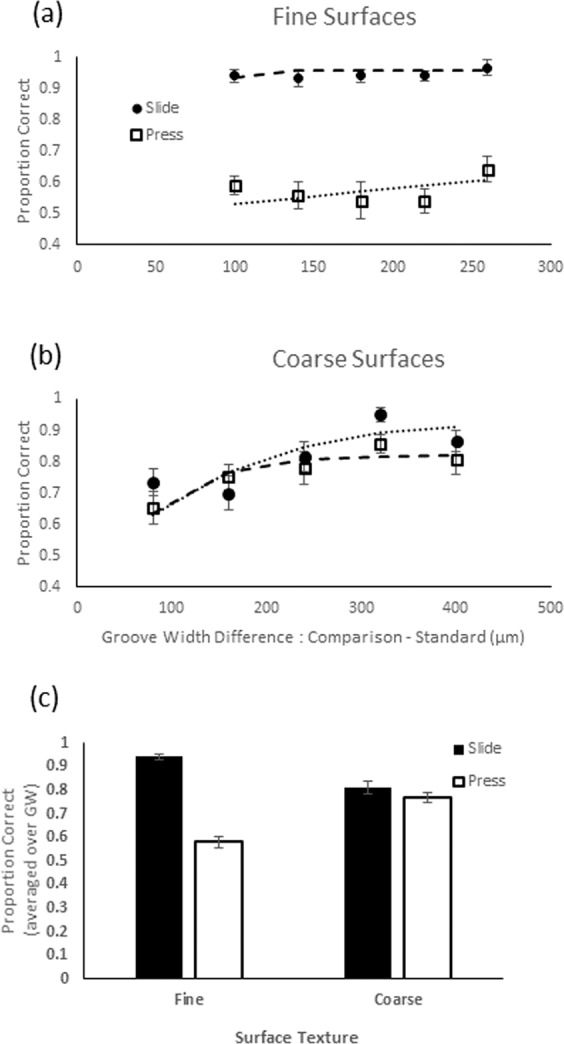
The proportion of correct responses when judging which of a pairs of (**a**) fine or (**b**) coarse gratings was the rougher. The groove widths (GW) of standard surfaces were 220 µm for the fine and 1120 µm for the coarse range. Pressing contacts with the surfaces are shown by the dotted lines with square markers. Sliding surface contacts are shown by dashed lines with circular markers. One SE of the mean is shown. Mean proportion correct (averaged over groove width) in each condition is shown in the bar graph (**c**) at the bottom of figure.

The average proportion of trials where the participant correctly reported which of the two stimuli on a trial was rougher is shown in Fig. 2(a,b) as a function of the difference in groove width between the standard and the comparison stimuli. The mean proportion of correct responses (averaged over groove width) in each condition is shown by the bar graphs in Fig. 2(c). A two-way repeated measures ANOVA to assess the effects of movement (pressing, sliding) and roughness range (fine, coarse) on the mean proportion of correct responses showed a main effect of movement type (F(1, 13) = 93.470, p < 0.01) and a significant interaction between movement and texture range (F(1,13) = 46.317, p < 0.01). Roughness discrimination was significantly worse when pressing on compared with sliding across fine surfaces (paired-sample t-test, t(13) = 14.295, p < 0.01) whereas there was no difference for coarse surfaces (t(13) = 1.126, p = 0.280).

### Contact forces summarised

**Figure 3:**
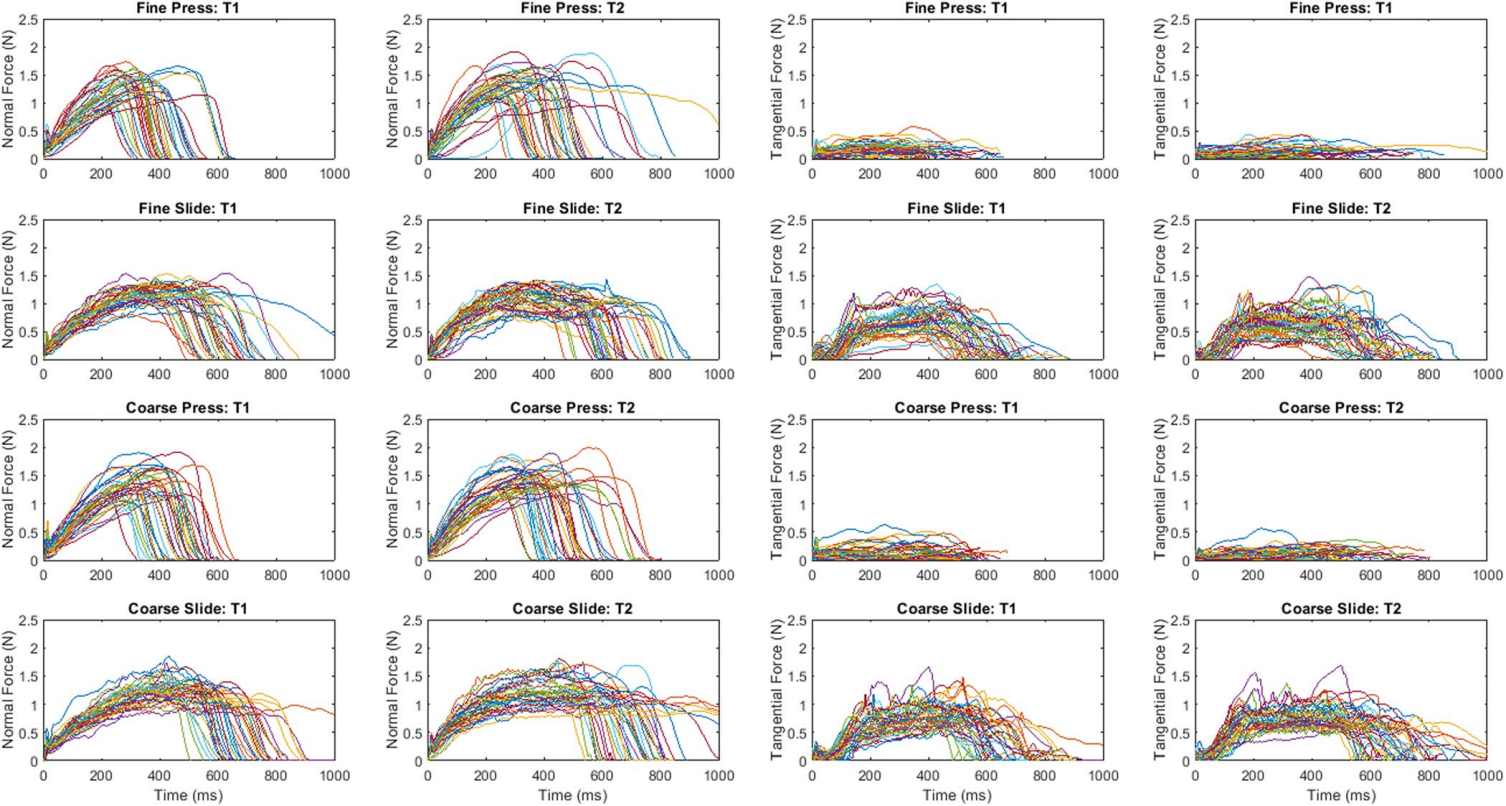
Illustrative normal (1^st^ and 2^nd^ columns) and tangential (3rd and 4th columns) force data for one participant. The force traces for different groove widths are overlaid. Force data from trials with fine surfaces are shown in the first 2 rows, those from coarse surface trials are shown in the final 2 rows. The touch on the first stimulus is labelled T1 and the second T2.

Illustrative contact force traces for a single participant are shown in Fig. 3. Participants’ target force was 1 N (windowed between 0.5 and 1.5 N). Contact duration was targeted at 1 second. The normal force was more single peaked and of shorter duration in pressing than in sliding conditions. Tangential forces recorded in pressing conditions were noticeably lower than those registered in sliding conditions. Force functions on touching the first and second stimulus were of similar form.

Across all participants average contact times with the fine and coarse surfaces were somewhat shorter than the 1 s target duration. Two-way repeated measures ANOVA (texture, movement type) revealed that sliding movements lasted on average 743 (SD = 171) ms, 32% longer than the 564 (SD = 215) ms duration of pressing movements (F(1,13) = 12.344, p < 0.01). Contacts with coarse surfaces lasted 667 (SD = 211) ms compared to fine surface contact time of 641(SD = 217) ms; a reliable main effect of surface showed this 4% difference was significant (F(1,13) = 11.152, p < 0.01). There was no significant interaction effect on contact duration between movement and surface. Across participants, the average normal force (during the middle 60% of the contact period) was close to the 1 N target. For sliding it was 1.032 (SD = 0.117) N and that for pressing was 1.180 (SD = 0.115) N, an 8% difference. Normal forces with coarse surfaces were 1.08 (SD = 0.114) N, only 3% higher than those for fine surfaces, 1.05 (SD = 0.113) N. Two-way ANOVA (texture, movement type) showed the main effect of sliding vs pressing was significant (F(1, 13) = 24.737, p < 0.001), as was the main effect of fine vs coarse (F(1,13) = 8.131, p = 0.013.There was no interaction between texture and movement.

### Relations between contact forces and accuracy

In the following sections normal and tangential force measures are each reported and analysed in terms of averages across median values for each participant. The initial analysis of each measure was a two-way repeated measures ANOVA with correct vs incorrect response in the discrimination task (accuracy) and sliding vs pressing movement (movement). As summarised in the Supplementary Materials, normal and tangential forces were largely unchanged as a function of groove width and first vs second touch and so the accuracy results are collapsed across these two factors for the majority of analyses.

### Normal force - mean

**Figure 4:**
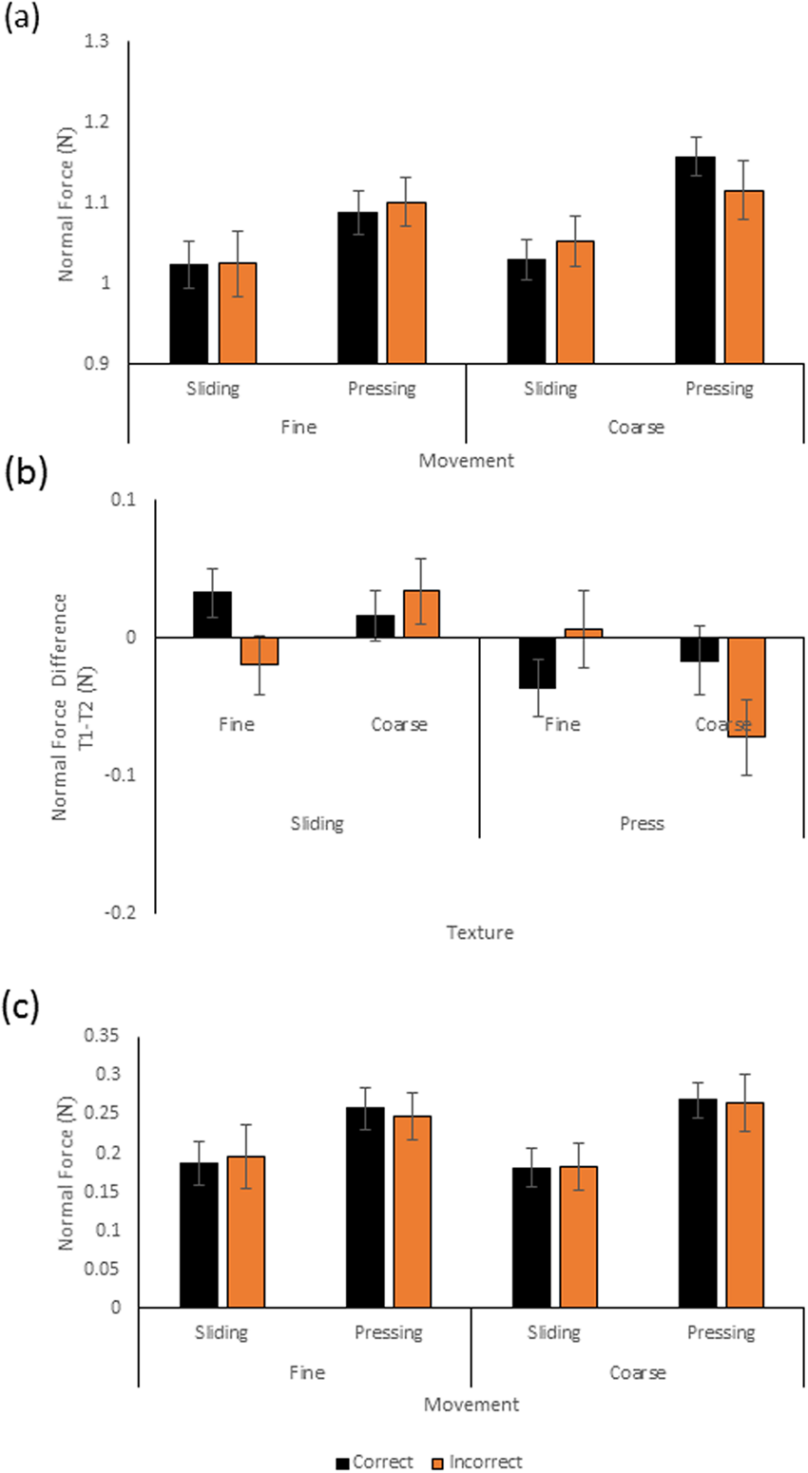
(**a**) The average mean normal force produced by participants on trials where they explored fine or coarse surfaces using sliding or pressing movements. The black columns represent mean normal force from trials where a correct roughness judgment was made. Orange columns represent averaged mean forces where the wrong perceptual response was given. (**b**) Difference in mean normal force between touch 1 and touch 2 as a function of surface and accuracy. One SE of the mean is shown. (**c**) The variability, in terms of standard deviation (SD), of normal force during a trial as a function of movement, surface texture range and accuracy. Black columns represent SD of mean normal force from trials with correct roughness judgments, orange columns SD of mean forces where the wrong discrimination response was given. One SE of the mean is shown.

The mean normal contact force as a function of texture, movement and accuracy is shown in Fig. 4(a). Two-way ANOVA of the fine data revealed no significant main effects or interactions, (all p > 0.118). Two-way ANOVA of the coarse data showed a main effect of movement (F(1,13) = 11.795, p = 0.004) and a significant interaction between movement and response accuracy (F(1,13) =5.713, p = 0.033). Correct judgments during pressing had significantly higher normal forces than those during sliding, (t(13) = −4.654, p < 0.001). There was no such difference for incorrect judgments (t(13) = −2.012, p > 0.065).

To further investigate the relation between accuracy and normal force in pressing on different surfaces, the difference in mean normal force between Touch 1 and Touch 2 on each trial was calculated. These data are shown as a function of texture, and response accuracy in Fig. 4(b). A two way ANOVA (with texture and accuracy as factors) of the coarse data for pressing showed a reliable interaction between texture and accuracy (F(1,13) = 11.537, p = 0.005). Force differences between intervals making up a trial were greater for incorrectly than correctly judged trials for coarse (t(14) = 2.193, p = 0.047)) but not fine (p = 0.082) surfaces.

### Normal force – within-Trial variability

The mean within-trial variability in normal force is shown in Fig. 4(c) in terms of standard deviation (SD). Separate two-way repeated measures ANOVAs for the fine and coarse data revealed higher variability with pressing than sliding contacts ((F(1,13) = 8.377, p = 0.013, for the fine and F(1, 13) = 12.153, p = 0.004, for the coarse data). There were no other significant main effects or interactions.

### Tangential force – mean

**Figure 5:**
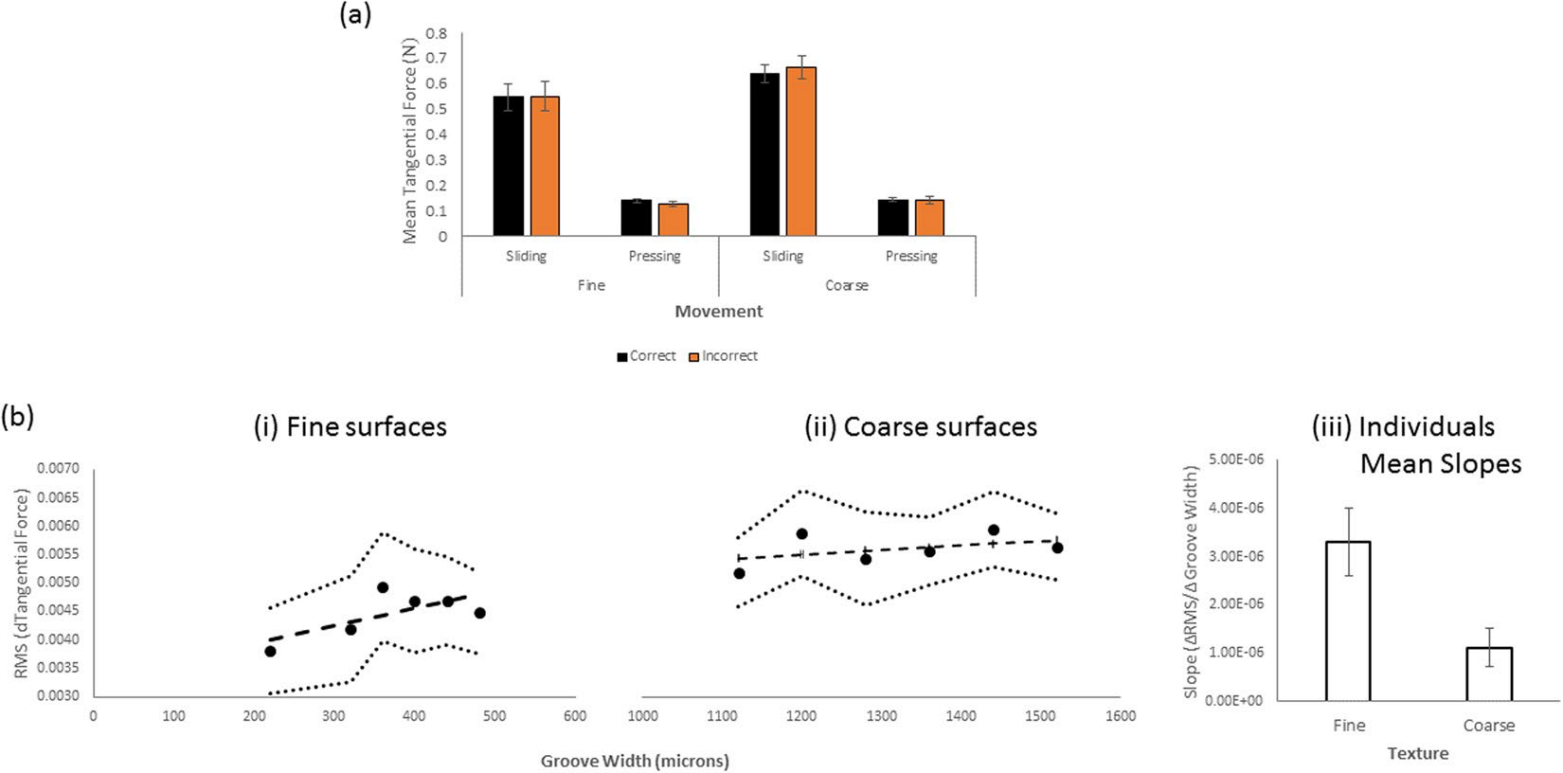
(**a**) Mean tangential sliding force for contacts as a function of movement, texture range and response accuracy. Data from trials with correct roughness judgments are shown by the black columns while forces from trials with incorrect roughness judgments are shown in orange. Error bars show 1 SE of the mean. (**b**) Differentiated tangential force RMS for sliding contacts as a function of groove width (µm) for surfaces in fine (i) and coarse (ii) ranges. The mean regression slopes across participants for fine and coarse texture ranges are shown in (iii). 1 SE of the mean in shown.

The averaged tangential force in sliding contacts with coarse and fine surfaces as a function of response accuracy can be seen in Fig. 5(a). Separate two-way repeated measures ANOVA of the fine and coarse mean tangential data revealed main effects of movement for the fine (F(1, 13) = 59.293, p < 0.01) and coarse stimuli (F1,13) = 122.269, p < 0.01). Both fine and coarse surfaces showed no main effect of discrimination accuracy nor any interactions with movement, F(1,13) ≤ 1.471, p ≥ 0.247.

### Tangential force - variability

Within-trial tangential force variability was quantified in terms of the root mean square (RMS) of the first derivative of the tangential force. Averaged RMS of the present tangential force data is shown as a function of groove width in Fig. 5b(i,ii). Mean slopes for individual participants, obtained using least-squares linear regression, are shown in Fig. 5b(iii). Slopes, describing increases in RMS with increases in groove width, were positive and significantly different from zero, t(13) = 5.690, p < 0.01 for fine and t(13) = 2.898, p = 0.012 for coarse surfaces. The average slope was significantly lower for coarse than fine surfaces, t(13) = 3.567, p = 0.003.

**Figure 6:**
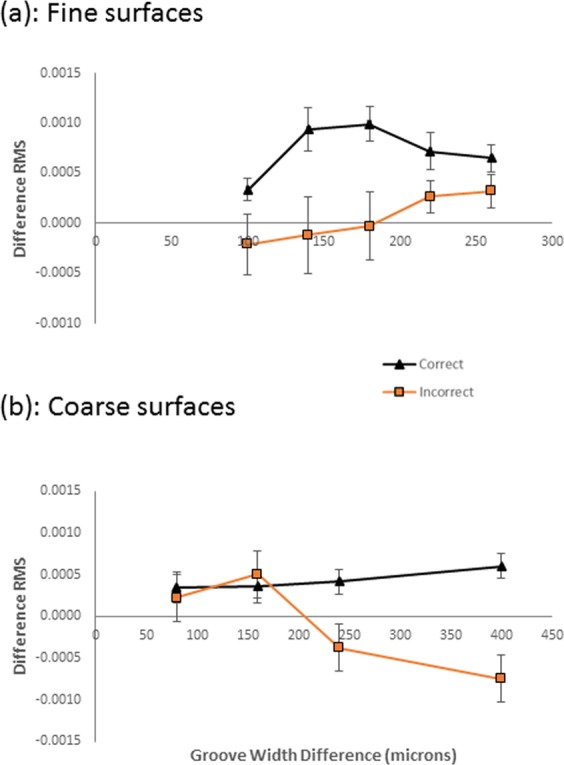
Comparison – standard tangential force RMS as a function of groove width and accuracy for fine (i) and coarse (ii) texture ranges. Trials with correct discrimination responses are shown in black. Those with incorrect responses are shown in orange. 1 SE of the mean is shown.

We calculated differences in tangential force fluctuations between the comparison and standard stimuli in each trial irrespective of whether each was the first or second surface touched on each trial. We examined whether these differences in tangential force variations, captured by the RMS, were different for correct compared with incorrect responses. The data are shown in Fig. 6. Separate two-way repeated measures ANOVAs (with accuracy and groove width as factors) for coarse and fine surfaces revealed RMS differences between the standard and comparison were greater on correct than incorrect trials (F(1,13) =12.430, p = 0.004 for fine stimuli and F(1,13) = 8.643, p = 0.011 for coarse. There were no other main effects or interactions.

## Discussion

In this paper, the accuracy of performance and contact forces (contact duration, normal and tangential force) have been analysed during a roughness discrimination task when pressing and sliding on ridged textures. The textures were regular 1-d spatial gratings with well-defined spatial periods in fine (320–580 micron) and coarse (1520–1920 micron) ranges. Participants attempted to keep peak force within a 0.5–1.5 N range and contact duration centred on 1 s.

The discrimination results replicate and extend previous research^8,19^, showing movement related differences in active texture perception depend on the type of texture being explored. We found roughness discrimination when sliding was better than when pressing onto surfaces in the fine but, importantly, not in the coarse range. This contrast between fine and coarse ranges is consistent with the predictions of the duplex theory of roughness discrimination^4,10^. With fine gratings the surface topography is likely insufficient to excite the SA mechanoreceptor channel during pressing contacts. In contrast, differences in vibrations set up by tangential force fluctuations during sliding contacts may be assumed to excite the PC channel - likely the primary source of roughness information under such conditions. In keeping with this view, we found poor performance in pressing and good performance in sliding contacts with fine gratings. With coarse gratings, the information available from the SA channel, whether pressing or sliding, appears to be sufficient for roughness discrimination with very similar performance for sliding over and pressing on coarse textures.

Analysis of contact forces showed contact duration was 30% longer for sliding than pressing. One possible interpretation of this effect is that, when participants attempted to control the duration of the sliding, they did not allow for dynamic phases of contact when movements were accelerating and decelerating. Contacts lasted slightly longer with fine than coarse textures. There were no consistent differences in duration as a function of response accuracy.

While mean normal force did not vary within a roughness range (see Supplementary Materials) as previously reported, See^15^ for a similar finding,  normal force was elevated on surfaces in the coarse compared to the fine range. This contrasts with a previous report^17^ comparing exploration of coarse versus fine surfaces. The latter’s use of aversive sandpaper stimuli, whose sharp particles tend to catch and abrade the skin, may account for their finding of reduced force with coarse compared with fine sandpaper. In the present experiment, we also found mean normal force was higher in pressing than sliding. Whereas normal force in sliding matched the 1 N target, in pressing it was some 18% higher than the target.

Correct discrimination responses were linked to greater normal force in pressing in the coarse but not the fine range. In general, the normal force on touch 1 was the same as on touch 2 regardless of accuracy. However, in coarse pressing, an increase in normal force on second compared to first touch was associated with incorrect discrimination. These effects were not evident in sliding. The improvement in performance due to force during pressing on coarse surfaces might be due to enhancement of the mechanical effects of the grating ridges and troughs producing greater activation of the slowly adapting mechanoreceptors. This interpretation might also explain why normal force in pressing was consistently higher than in sliding.

Variability of normal force was reliably higher in pressing than in sliding. However, the accuracy of discrimination was unrelated to variability. Normal force results in a frictional force during sliding that opposes the tangential force producing the sliding. It might have been thought that variability in tangential force would produce less consistent sliding which, in turn, might have resulted in less accurate roughness discrimination. That this did not occur could be seen as consistent with the finding that roughness perception is unaffected by sliding velocity^20^.

The difference in variability of tangential force between standard and comparison stimulus was reliably greater for correct compared to erroneous responses in both fine and coarse ranges. This suggests that fluctuation in tangential force is a cue for roughness discrimination during sliding, consistent with the involvement of the PC channel. Such a possibility is further supported by the finding that tangential force variability exhibited a significant increase with groove width. A complementary result showing roughness magnitude estimates of spatial gratings increase with tangential force variability was noted by Smith *et al*.^14^.

In the present study participants were asked to produce forces ranging between 0.5 and 1.5 N. Previous studies have shown variability across participants in the contact forces voluntarily employed when evaluating surface roughness (Lederman, 1974, Meftah *et al*., 2000). Meftah and colleagues found instructions to adopt a “Comfortable contact force” during moving contacts elicited normal forces ranging from 0.79–1.41 N with a mean of ~1 N. Lederman (1974) reported the deployment of a somewhat wider, though overlapping, range (0.17–1.70 N with a mean of ~0.66 N) in response to instructions to using ‘normal’ contact forces. Following Meftah *et al*., our participants were asked to use contact forces in the range of 0.5 to 1.5 N. Our anticipation that the “comfortable contact force” of most of our participants would fall within this range was supported by our findings that normal forces in sliding conditions were around 1 N. The increased force used in pressing conditions (mean of ~1.1 N), suggests that range provided sufficient scope for variation in the contact forces. However, it is possible that the limit on normal force (as well on exploration time) might have constrained participants’ strategic use of force to optimise their discrimination performance. In future it will be interesting to investigate the removal of the experimenter-imposed constraint on normal force to see whether participants might choose a greater range of forces. In that case it might be that force differences observed in the present experiment would increase.

In conclusion we have examined the relationship between active force production, the form of contact made with a surface (pressing or sliding) and the impact these factors have on roughness discrimination. As predicted by the duplex theory of roughness discrimination, we show that contact forces vary systematically with task parameters and can affect discrimination accuracy in a manner that depends on the type of contact (sliding vs pressing) and the coarseness of the texture (fine vs coarse).

## Methods

The experiment was performed in accordance with the principles outlined in the Declaration of Helsinki. Ethical approval was provided by the Psychology Ethics committee at the University of Birmingham (ERN_09-528AP24). All of the participants gave written informed consent.

### Participants

Data were collected from 14 healthy participants recruited from the University of Birmingham, aged between 19 and 32 years (eight were female). Each participant received £15 for taking part. All participants were right hand dominant using the Edinburgh Handedness Inventory^21^.

### Experimental set-up

The experimental set-up and stimuli are shown in Fig. 1.

Roughness discrimination was assessed using 2 sets of Tufset polyurethane gratings (35 mm × 29.5 mm), all machined using computer numerical control (CNC). Each set of gratings (a coarse and a fine set) comprised 1 standard stimulus (SS) and 5 comparison stimulus (CS) surfaces. The details of the surfaces are shown in Table 1.

On each trial, pairs of stimuli from the fine or coarse range set were held securely in a machined metal plate (see Fig. 1) mounted on a 6 degree of freedom force-torque transducer (ATI Nano 43, NC, USA). A small metal dome at the far side of each grating allowed the participant to locate the next grating to be touched. The transducer had a resolution of 0.002 N for the forces (Fx, Fy, Fz) and 0.025 Nmm for the Torques (Tx, Ty, Tz). The data were acquired at a sampling rate of 1 kHz and were filtered using a second order, low pass Butterworth filter with a cut-off frequency of 100 Hz.

### General Procedure

The participants’ tactile abilities were quantified by their performance in two ancillary tasks (grating orientation test, GOT and dexterity test; see Supplementary Materials) and the roughness discrimination task. We also measured the right index finger pad surface area across the lateral border from the fingertip to the distal interphalangeal joint using a set of callipers. The data were collected in two 90 minute sessions carried out on separate occasions separated by between 3 and 10 days. The ancillary tasks and finger area measurement were carried out in the first half of session 1. Participants completed half (80 trials) of the roughness discrimination task during the rest of the session. The remaining 80 trials making up the discrimination task were conducted at the start of session 2.

### Roughness discrimination

The participants actively explored pairs of grating stimuli drawn from the same range (fine or coarse) and indicated which of the pair, presented in a random order, was rougher. At the start of the experiment the participants used a cleansing wipe (https://www.pampers.co.uk) to remove any dirt or grease from their skin. They sat at a desk on which the force sensors and grating stimuli were placed.

The session began with a training period in which participants practiced applying between 0.5 N and 1.5 N of normal force while stroking the gratings in an anterior-to-posterior direction (sliding condition) or pressing onto the stimulus surfaces (pressing condition). Force levels were selected following comfortable force levels chosen by participants in pilot testing, in combination with the finding of a mean force of 0.7 N during freely chosen force during roughness exploration^11^. Visual feedback of the applied force was given on a computer screen. Training was continued until participants were able to produce the correct force on 3–4 consecutive trials.

The roughness discrimination task commenced following training. At the start of each discrimination trial participants positioned their right index finger against a smooth metal dome projecting from a cover above the force sensor. Participants were then cued by a series of 3 beeps, the last of which was highest in pitch and indicated that the participants should begin to explore the surface below. The participants then either stroked or pressed on the grating using the correct force, before placing their finger against the dome positioned over the second stimulus surface. This surface was explored in a similar manner following the auditory cues. Participants were asked to maintain contact with the stimulus surface for approximately 1 s. Trials with exploratory forces outside the expected range were repeated until a permitted force was used. Participants gave a verbal response indicating whether the first or second of the explored surfaces was the rougher of the pair. No feedback was given about discrimination judgments. The participants were blindfolded and white noise played over headphones masked auditory cues arising from finger contact with the stimulus surfaces.

There were 160 roughness discrimination trials. On half of these trials the participant was instructed to press their finger (applying a normal force while minimising sliding movements) onto the grating surface. On the remaining 80 trials participants were instructed to slide their finger across the gratings. Of the 80 trials in each experimental session, half involved sliding movements and half pressing movements. These trials were run in a blocked design with all of the trials from one movement condition tested before the other condition began. Half of the participants began with pressing trials, and half began with sliding trials. Fine and coarse discrimination pairs occurred in every block of trials with their order randomised. Each pair of stimuli was presented 8 times in total. Participants took breaks every 20 trials.

### Data processing of contact forces

The data were processed using custom designed Matlab (https://www.mathworks.com/products/matlab.html) scripts. The onset and offset of contact between the finger and the textures was estimated from changes in normal force. Mean and standard deviation of force readings were calculated for the 2 seconds immediately prior to an auditory cue instructing the participants to touch the first of the pair of textured surfaces. The first normal force value 4 standard deviations above this mean noise, with 200 subsequent readings above (below) this threshold was recorded as the onset (offset) of contact. Values found using this algorithm were visually verified on each trial. The contact onset and offset values derived for the first and second touches in a trial were used to calculate the length of time participants’ maintained contact with the stimulus surfaces (the contact duration).

Normal and tangential forces were characterised by mean and standard deviations (SDs) of values calculated over the mid-60% of the contact sections of the force trace. This time window ensured that the summary statistics reflected forces when the finger was securely in contact with the stimulus surface, avoided transient changes in force associated with the onset and offset of finger-surface contact and, in the sliding conditions, described motion across the surfaces. Median values of the normal and tangential force means and SDs were used to characterise performance in each condition for each participant. In conditions where no roughness discrimination errors occurred (on average this was less than 4% of all the trials in those conditions) null entries were replaced using stochastic regression imputation methods^22^ in SPSS.

## Supplementary information

Supplementary information

## Data Availability

The data will be made available on acceptance for publication.
