# Supplementary material for: Contact forces in roughness discrimination
Source: Sci Rep. 2020 Mar 20;10:5108. doi: 10.1038/s41598-020-61943-x (PMC7083896; doi:10.1038/s41598-020-61943-x)
Supplement: Supplementary file 1 — Supplementary information [file 41598_2020_61943_MOESM1_ESM.pdf]

Contact forces in roughness discrimination

Supplementary materials

Roberta D Roberts[1]*, Al R Loomes[1], Harriet A Allen[2], Massimiliano Di Luca[1] and Alan M Wing[1]

[1]School of Psychology, University of Birmingham, [2] School of Psychology, University of Nottingham

Correspondence should be addressed to R. Roberts. (email: R.Roberts@bham.ac.uk)

Ancillary Tactile Tasks.

Tactile spatial acuity was measured using a set of grating domes with equidistant groove and ridge widths [range: 0.35mm, 0.5mm, 0.75mm, 1.00mm, 1.25mm, 1.5mm, 2.00mm, and 3.00mm] (JVP Domes, Stoelting, Inc.). Manual dexterity of the right hand was assessed using speeded completion of a nuts and bolts test. This test comprised a wooden board containing five fixed bolts and five nuts in adjacent, recessed squares. The nuts could be screwed onto and unscrewed from the corresponding nut.

Grating Orientation Task.

The grating orientation task (GOT), a widely used test of spatial sensitivity E.g. [1,2-4], was used here on the right index finger. One of the grating domes was applied to the glaborous skin of the distal phalanx of the finger for approximately 1-2s with a medium force (of around 1-2 N). The orientation of the grating, grooves parallel or transverse to the long axis of the finger, was randomly determined on a trial-by-trial basis. Participants were asked to report the groove orientation across the finger. The GOT session began with a brief training period (6 trials) using the dome with the widest groove width (3mm). Participants were informed about the orientation of the grooves following the first five contacts between the dome and skin. On the sixth practice trial, the participants were asked to report the groove orientation and were given feedback about their response. Participants were then tested with the whole set of GOT domes. Each dome was pressed onto finger pad for 20 times and an orientation judgement sought after each contact. The dome presentation order went from largest groove width (3mm), to smallest (0.35mm). The threshold for each participant was

calculated by finding the smallest groove width with which orientation could be correctly discriminated 75% of the time. Participants wore a blindfold throughout the GOT task.

Nuts and Bolts Test (Manual Dexterity).

The participants sat at a table with both hands placed flat on the surface and the nuts and bolts board placed in front of them (see figure 5). They were first instructed to screw all the nuts on the bolts, using only their right hand, as quickly as possible. They were informed that each of the nuts had to be screwed right down to the bottom of each of the bolts. Following the participant's completion of this part of the task, the experimenter manually ensured that all the nuts were at the bottom of the bolts (i.e. no further movement was possible).The participants were then asked to use their right hand to unscrew each of the nuts from the bolts, placing them back in the original holes as quickly as possible. The time taken to perform the screwing and unscrewing actions was recorded.

Results

Ancillary Measures.

We performed a series of measure to capture the participants' physical characteristics, sensory and motor abilities. Mean score on a grating orientation test (GOT), a measure of (static) tactile spatial resolution was 1.84 mm (SD=0.83 mm). The average index finger pad skin area was 320 m^2 (SD=0.55mm). Finger GOT threshold and finger surface area was positively correlated with ($r(11) = 0.718$, $p=0.013$, measures on 3 participants were not taken). Dexterity was quantified by asking participants to first screw and then unscrew nuts

on a set of 6 fixed bolts and measuring completion time for the two tasks, which were 31.56 s (SD = 6.34) and 26.10 s (SD = 6.20).

Correlations between normal forces and ancillary measures

There were no significant correlations between ancillary measures and discrimination performance. Nor whether there any significant correlations between ancillary measures and the mean normal forces in any of the experimental conditions. This latter result replicates previous findings of a lack of association between roughness perception and mean normal forces (Libouton et al., 2010; Smith, Chapman et al., 2002). There was a negative correlation between GOT threshold and the T1 – T2 force difference when pressing on fine surfaces in incorrectly judged trials (Pearson's $r(14) = -0.665$, $p = 0.009$). Participants with lower spatial sensitivity press harder on the second compared with first contact interval. There were no other significant correlations. The data also replicate a previous [5] of an association between low spatial sensitivity and larger finger size ($r(11) = -0.718$, $p = 0.013$).

Effects of groove width

The following section describes contact force measures as a function of groove width , texture range (fine vs coarse), contact interval in a trial (1st vs 2nd), and movement (sliding vs pressing).The coarse and fine surfaces used different groove width ranges and were therefore analysed separately using 3-way ANOVAs with groove width, touch and movement as factors.

Contact Duration

The mean contact times between participants' fingers and the stimulus surfaces were averaged across participants and are shown in figure SM1 as a function of groove width.

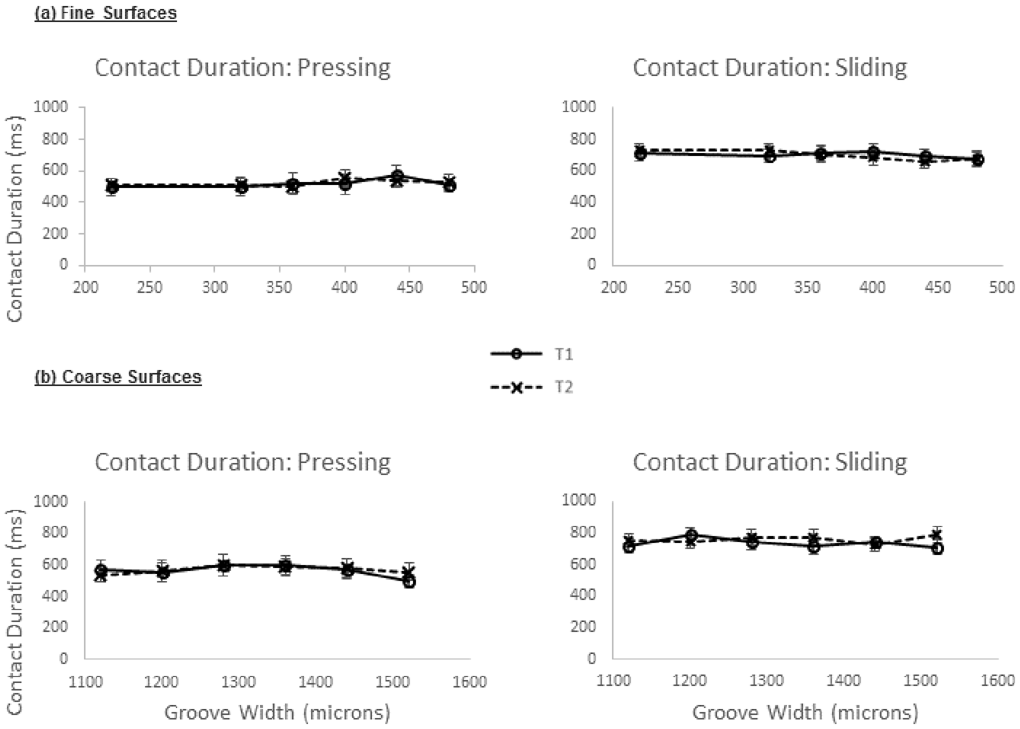

Figure SM1. The mean duration of contact (in ms) between participants' fingers and the textured stimuli (calculated from normal force). The length of contact with fine surfaces is shown by (a), while contact with coarse surfaces is shown by (b). Pressing and sliding contacts in the first (T1) and second (T2) intervals of a trial along with 1 SE of the mean are shown.

As is evident in figure SM1, the participants touched the fine surfaces for longer when sliding across compared with pressing on them, ($F(1,13)= 31.456$, $p<0.001$). A significant interaction between movement condition and GW ($F(5,65)= 4.485$, $p= 0.001$) was specific to pressing contacts ($F(5,65) = 3.259$, $p=0.011$). Contact times with the 400 and 440 µm were longer than for the remaining surfaces. An ANOVA excluding this former pair of GW's showed no systematic difference on contact time with GW ($F(3,39) =0.676$, $p=0.572$).

Contact times were also longer for sliding than pressing contacts with coarse surfaces, ($F(1,13)= 23.459$, $p <0.001$), with no other main effects or interactions.

Normal force - mean.

The mean normal forces participants exerted on the textures of different groove widths are shown in Figure SM2.

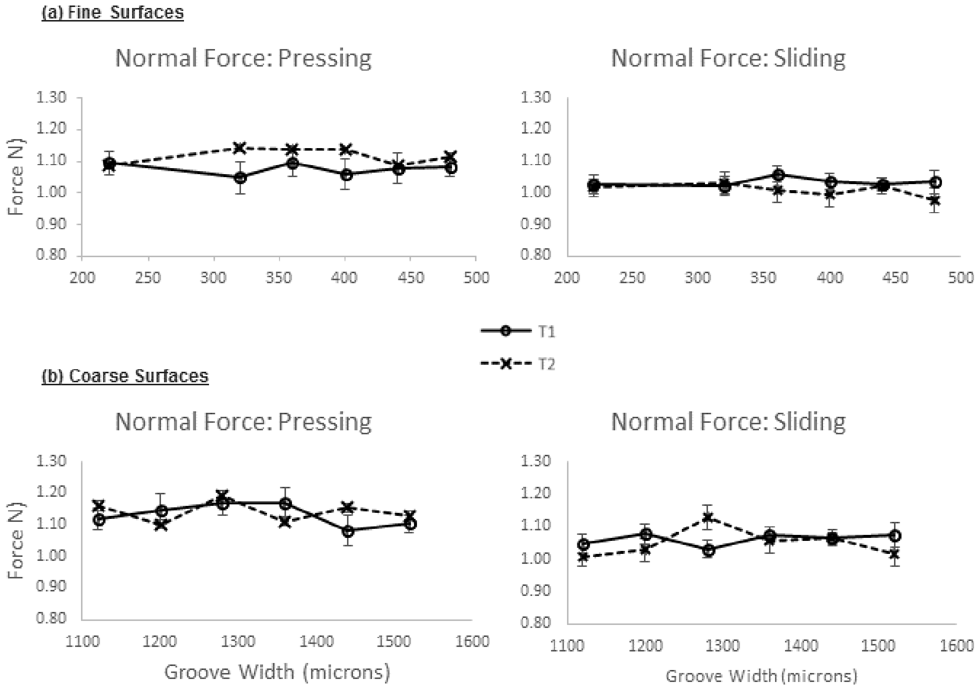

Figure SM2: The normal force at each grating groove width. Mean forces from the first interval of trials (T1) are shown as open circles. Second interval (T2) normal forces are shown as x's. The values shown were averaged over participants. The forces used when touching fine surfaces are shown at the top of the figure, while those for contact with coarse surfaces are shown at the bottom. One SE of the mean is shown by the error bars.

Three-way ANOVA for fine surfaces showed higher forces for pressing than sliding ($F(1, 13) = 6.665$, $p=0.023$) but only for the second contact interval, interaction between movement and contact interval, ($F(1, 13) = 8.475$, $p=0.012$). The same analysis for coarse data showed higher mean normal forces pressing than sliding, ($F(1, 11) = 11.505$, $p= 0.006$) with no other main effects or interactions for either coarse or fine surfaces.

Normal force - variability.

The within trial SD of normal force is shown in Figure SM3. Analysis of the fine data showed an effect of movement (F1, 13) = 10.342, $p=0.007$) reflecting elevated SD in pressing compared to sliding. There were no other main effects or interactions. The analysis of coarse data revealed a similar pattern of behaviour. Forces were more variable for pressing than sliding movements across coarse surfaces, $F(1, 11)= 11.149$, $p=0.007$. An interaction between movement and contact interval ($F(1,13)= 5.794$, $p=0.032$) reflected increased variability in the second compared with first contact interval for pressing but not for sliding touches.

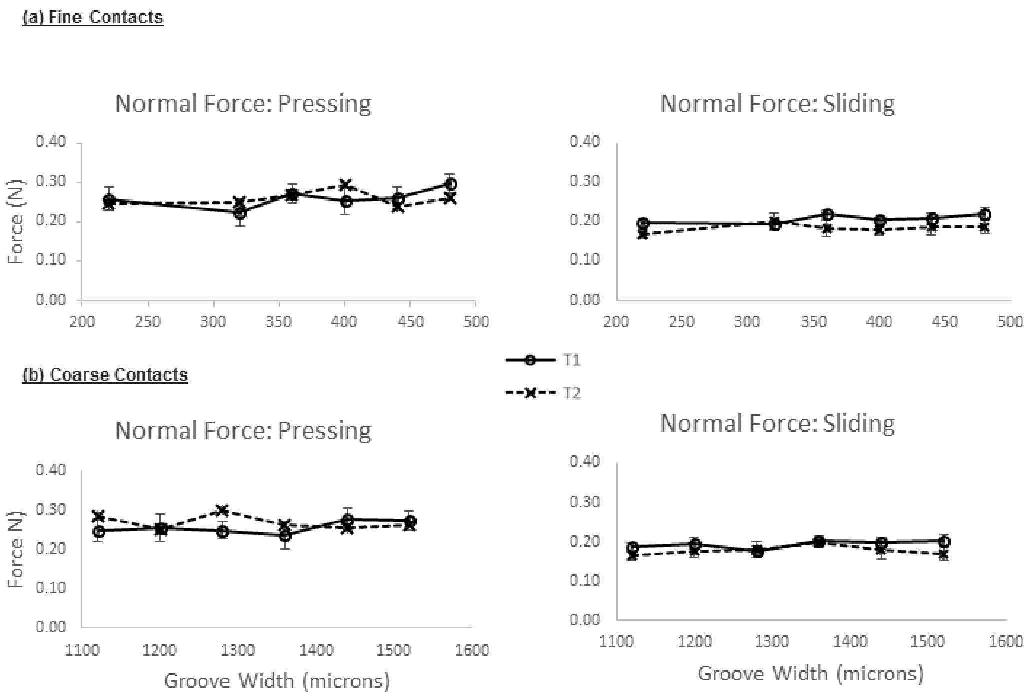

FigureSM3: The standard deviation of normal force during a trial. Values for the first contact interval are shown in open circles, the second interval by x's. Data are shown for trials from each groove width. One SE of the mean is shown.

Tangential force - mean.

The averaged tangential force in sliding contacts with coarse and fine surfaces can be seen in Figure SM4. For fine surfaces a two-way ANOVA revealed a main effect of touch, with higher tangential force on the first compared to the second touch ($F(1,12)= 39.128$, $p<0.0005$). The same pattern of higher mean tangential force on touch 1 than touch 2 was observed for the coarse data ($F(1, 12)= 8.030$, $p=0.015$). Analysis of the coarse data also revealed a significant effect of GW ($F(5,65)= 5.938$, $p<0.001$) resulting from a significantly higher tangential force with the 1120 μm standard than other coarse surfaces. Analysis of the data without the 1120 μm surface showed no effect of GW ($F(4,52)= 0.772$, $p=0.548$).

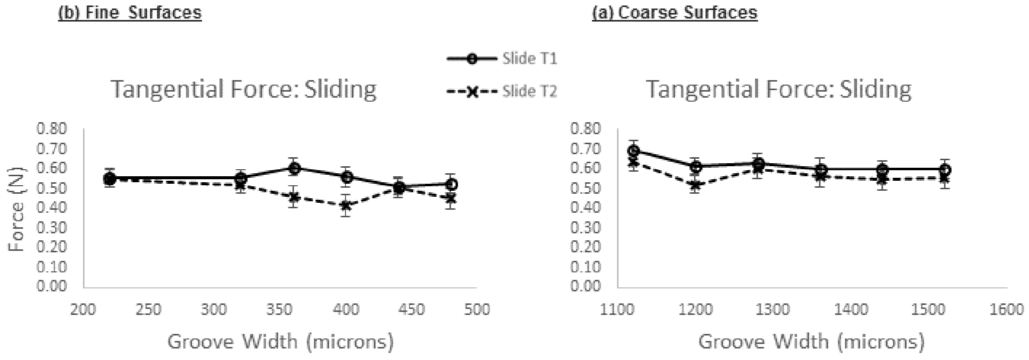

Figure SM4: Tangential force in sliding contacts with fine and coarse surfaces; *Values for the first contact interval are shown in open circles, the second interval by x's. Data are shown for trials from each groove width. One SE of the mean is shown.*

Dynamic Coefficient of Friction

The ratio of normal force to tangential force during sliding is a measure of the dynamic coefficient of friction (dCOF). This ratio was estimated for each trial and averaged over repetitions and contact intervals. The slopes and intercepts of linear functions fitted to these data were optimised using MS Excel's Generalized Reduced Gradient routine. dCOF showed small linear increases with groove with for both coarse and fine surfaces (see SM5).

The sloped of these functions were no different from each other (t(13)=-0.136, p=0.894).

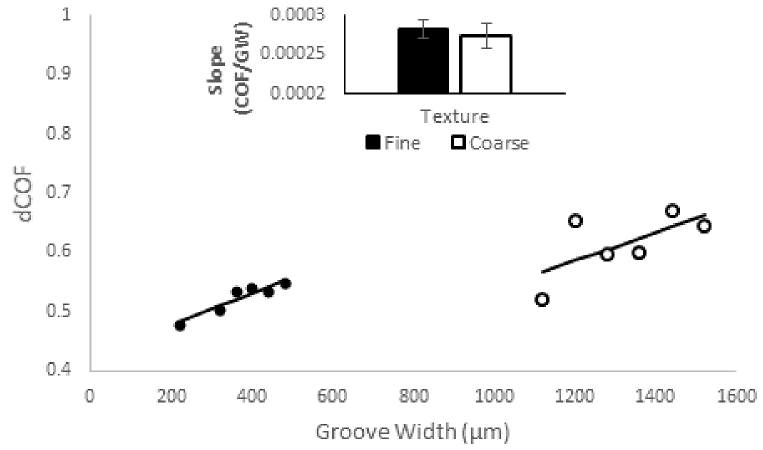

Figure SM5: The line graphs show the mean dynamic coefficient of friction (dCOF) for each groove width. Fine surface data are shown by filled circles, coarse data by unfilled circles. Mean slope of linear functions fitted to individual data are shown by the bar graph insert. *One SE of the mean is shown.*

References

1 Bleyenheuft, Y. & Thonnard, J. L. Tactile spatial resolution measured manually: A validation study. *Somatosens Mot Res* **24**, 111-114, (2007).

2 Norman, J. F. *et al.* Aging and the Haptic Perception of Material Properties. *Perception* **45**, 1387-1398, (2016).

3 Norman, J. F. *et al.* Aging and Curvature Discrimination from Static and Dynamic Touch. *Plos One* **8**, (2013).

4 Vanboven, R. W. & Johnson, K. O. The Limit of Tactile Spatial-Resolution in Humans - Grating Orientation Discrimination at the Lip, Tongue, and Finger. *Neurology* **44**, 2361-2366, (1994).

5 Peters, R. M., Hackeman, E. & Goldreich, D. Diminutive digits discern delicate details: fingertip size and the sex difference in tactile spatial acuity. *J Neurosci* **29**, 15756-15761, (2009).
